# The Skeletal Muscle Emerges as a New Disease Target in Amyotrophic Lateral Sclerosis

**DOI:** 10.3390/jpm11070671

**Published:** 2021-07-16

**Authors:** Oihane Pikatza-Menoio, Amaia Elicegui, Xabier Bengoetxea, Neia Naldaiz-Gastesi, Adolfo López de Munain, Gorka Gerenu, Francisco Javier Gil-Bea, Sonia Alonso-Martín

**Affiliations:** 1 Neuromuscular Diseases Group, Neurosciences Area, Biodonostia Health Research Institute, 20014 Donostia/San Sebastián, Spain; oihane.pikatza@biodonostia.org (O.P.-M.); amaia.elicegui@biodonostia.org (A.E.); xabier.bengoetxea@biodonostia.org (X.B.); neia.naldaiz@biodonostia.org (N.N.-G.); ADOLFOJOSE.LOPEZDEMUNAINARREGUI@osakidetza.eus (A.L.d.M.); gorka.gerenu@biodonostia.org (G.G.); francisco.gilbea@biodonostia.org (F.J.G.-B.); 2 CIBERNED, Carlos III Institute, Spanish Ministry of Economy & Competitiveness, 28031 Madrid, Spain; 3 Department of Neurology, Donostialdea Integrated Health Organization, Osakidetza Basque Health Service, 20014 Donostia/San Sebastián, Spain; 4 Department of Neurosciences, Faculty of Medicine and Nursery, University of the Basque Country UPV-EHU, 20014 Donostia/San Sebastián, Spain; 5 Department of Physiology, University of the Basque Country UPV-EHU, 48940 Leioa, Spain

**Keywords:** ALS, skeletal muscle, neuromuscular junction, metabolism, vesicles, distal axonopathy, neuromuscular disorder

## Abstract

Amyotrophic lateral sclerosis (ALS) is a fatal neurodegenerative disorder that leads to progressive degeneration of motor neurons (MNs) and severe muscle atrophy without effective treatment. Most research on ALS has been focused on the study of MNs and supporting cells of the central nervous system. Strikingly, the recent observations of pathological changes in muscle occurring before disease onset and independent from MN degeneration have bolstered the interest for the study of muscle tissue as a potential target for delivery of therapies for ALS. Skeletal muscle has just been described as a tissue with an important secretory function that is toxic to MNs in the context of ALS. Moreover, a fine-tuning balance between biosynthetic and atrophic pathways is necessary to induce myogenesis for muscle tissue repair. Compromising this response due to primary metabolic abnormalities in the muscle could trigger defective muscle regeneration and neuromuscular junction restoration, with deleterious consequences for MNs and thereby hastening the development of ALS. However, it remains puzzling how backward signaling from the muscle could impinge on MN death. This review provides a comprehensive analysis on the current state-of-the-art of the role of the skeletal muscle in ALS, highlighting its contribution to the neurodegeneration in ALS through backward-signaling processes as a newly uncovered mechanism for a peripheral etiopathogenesis of the disease.

## 1. Introduction

Neuromuscular diseases involve the injury or dysfunction of peripheral nerves and muscles [1]. Indeed, these disorders affect the efferent nerve fibers that control voluntary muscles and those communicating sensory information back to the brain. When motor neurons (MNs) become dysfunctional or die, communication between the central nervous system (CNS) and muscle tissue breaks down, resulting in muscle weakness and wasting (atrophy) [2]. Amyotrophic lateral sclerosis (ALS) is included within this broad group of disorders, though also associated with CNS diseases. In fact, ALS is characterized by progressive degeneration of MNs in the brain and the spinal cord, which control the contraction of muscles that enable moving, speaking, breathing, and swallowing. Despite being the most common degenerative MN disease, ALS is classified as a rare disease based on its low prevalence due to its high mortality; the survival rate is relatively short, between 2 and 5 years on average [3], placing ALS as one of the most devastating diseases among all deadly disorders. Current treatments, such as the glutamate antagonist Riluzole, or the free radical scavenger Edaravone (only approved in few countries), slow down disease progression but are unable to reverse nerve damage or muscle weakness [4,5].

Overall, ALS is considered a fatal MN disease, coursing with progressive degeneration and death of upper and lower MNs, severe muscle atrophy, respiratory distress, and cellular protein aggregation [6]. Most ALS forms appear sporadically (sALS), whereas only 10% of ALS cases, considered as familial (fALS), involve an identifiable and heritable genetic component, typically acting in an autosomal dominant manner. Nonetheless, the primary cause of ALS remains unknown in the majority of the cases. Over 30 genes have been linked to ALS thus far [7], with the most prevalent ALS-associated mutations being located in *C9ORF72* (chromosome 9 open reading frame 72), *SOD1* (Cu/Zn superoxide dismutase 1), *TARDBP* (TAR DNA-binding protein 43; *TDP-43*), and *FUS* (fused in sarcoma) [8]. Interestingly, most of the causative and susceptibility genes participate in particularly relevant cellular functions, such as DNA/RNA processing, autophagy, vesicle transport, oxidative stress, or metabolism [7]. External causative factors have also often been proposed for sALS, but none were totally confirmed, including exogenous environmental neurotoxins, heavy metals, dietary factors, physical exertion or trauma, or genetic factors [7,8]. It is worth noting that recently, arginine-rich cell-penetrating peptides have been associated with the onset of ALS, with a toxic effect linked to a widespread displacement of DNA- and RNA-binding factors [9].

Although ALS has been considered a neurodegenerative disease since its discovery, today it is defined as a multisystem disorder that includes changes in structural, physiological, and metabolic parameters in different cell types, which act mutually and synergistically contribute to the onset and severity of the disease [10,11]. Indeed, the recent growing number of clinical and animal/cellular studies provides unclouded evidence that MN damage could arise from *non-cell autonomous* mechanisms displayed by glia [12] or muscle cells [13]. In this line, growing molecular evidence supports the fact that MN diseases can also occur following distal axonal degeneration, supporting the “dying back” hypothesis, implying that pathological changes appear in the axon distally, at the NMJ or even in the skeletal muscle, and are transmitted into the soma prior to the onset of clinical symptoms and MN death [14,15]. The NMJ is a highly specialized synapse, which controls signaling between muscle and nerve that is necessary for skeletal muscle fitness. Notably, successful restoration of functional innervation (muscle-nerve interactions) during muscle repair is essential to preserve muscle motor function. Interestingly, the skeletal muscle shows a key role in these events by secreting specific factors during regeneration, postulating that attractive and repulsive signals used for axon guidance could be implicated in such a process [16].

Strikingly, specific overexpression of mutated *SOD1* in MNs does not drive ALS-like pathology, whereas specific *SOD1* modulation in mouse skeletal muscle leads to an ALS phenotype including muscle atrophy and MN degeneration [17,18]. Furthermore, the autopsy of an early-staged ALS patient demonstrated muscle changes with signs of muscle denervation and reinnervation, though the patient had healthy MNs [19]. All these pieces of evidence have reshaped the model proposing that, at least in some ALS cases, pathological changes may start in the skeletal muscle and spread through the neuromuscular junction (NMJ) prior to MN degeneration and the onset of clinical symptoms [14]. Indeed, the notion that MN damage could be secondary to muscle wasting and is not exclusive to the ALS condition but rather supported by evidence of MN loss in individuals with age-related sarcopenic muscle [20]. Common pathological features in ALS regarding MNs and muscle are indicated in Table 1. Taken together, these recent findings drag the skeletal muscle into the spotlight of ALS research.

In this review, we present an overview of published and ongoing studies highlighting peripheral muscular involvement in the development of ALS through NMJ “back-signaling” for MN degeneration. Finally, we also discuss the metabolic facet of the muscle itself, paving the way to a better understanding of the disease by accepting muscle signaling as a key contributor to ALS disease. Thus, tackling the skeletal muscle as a direct target in ALS disease could accelerate potential advances in future therapeutic interventions.

## 2. Motor Neuron Degeneration as *Non-Cell Autonomous* Process

Progressive MN degeneration is considered the main cause of ALS. However, to date, there are no effective disease-modifying therapies. This lack of success could have been triggered by the MN-centric perspective on ALS neurodegeneration (*cell autonomous*), holding back the relevance of ALS-related alternative mechanisms and limiting the emergence of new therapeutic strategies.

The relationship between MN degeneration and NMJ denervation in ALS remains elusive, but recent advances have demonstrated that NMJ functionality could play a crucial role in ALS development [21]. The homeostatic balance of MNs is especially refined due to their ATP-dependent axonal transport rate, axon length [22], and excitability pattern [23], demanding high support of adjacent cell populations under physiological and pathological conditions. Non-neuronal cells of NMJ (i.e., terminal Schwann cells and kranocytes) supply this nutritional, metabolic, and trophic supply, playing a key role in commandeering MN function and health (discussed in the NMJ section). Therefore, non-neuronal cells’ alterations could lead to insufficient support to MNs, affecting their homeostasis, functionality, and eventually, promoting neurodegeneration. Thus, recent findings have questioned the neurogenic origin (*cell autonomous* mechanism) of the disease, placing it on the NMJ´s non-neuronal cells (*non-cell autonomous* mechanism), such as glia or muscle [24]. MN degeneration has been associated with neuroinflammatory processes, which are starting to be considered part of the cause of neuronal damage and not just a consequence [25]. However, this inflammatory response has not been fully characterized in ALS. Non-neuronal cells can induce both protective and toxic effects on MNs under physiological and pathological conditions, respectively. Neuroinflammation leads to the proliferation and activation of astroglia, microglia, and oligodendrocytes, which secrete reactive oxygen species (ROS) and pro-inflammatory cytokines (such as TNF-α, IL1β, and IL6) [26]. Early in the 90s, the activation of microglia was already associated with the infiltration of Th and Tc cells in the spinal cord and the motor cortex of ALS patients [27]. Th cells activate astrocytes and microglia, switching them from a neuroprotective function to a neurotoxic one [28]. In addition, blood samples from patients and animal models of ALS showed low levels of regulatory T cells, which promote the neuroprotective phenotype in microglia [25,29]. Therefore, these pieces of evidence indicate that immune response, by becoming chronic, can contribute pathologically to the development of ALS.

Within the CNS, there is a large list of cell types classified as non-neuronal cells, which include astrocytes, microglia, oligodendrocytes, endothelial cells, and pericytes, among others. Although the activity of all of them is physiologically important within the CNS, some of them are particularly relevant in pathological conditions. More specifically, microglia and astrocytes have been further detailed in ALS pathology, which will be the focus of the following sections.

### 2.1. Microglia

This section discusses the relevance of metabolic and nutritional support provided by glia cell types to MNs and how tipping the homeostasis of these cells could affect MN physiology and lead to neurodegeneration. Microglia make around 10% of total glial cells and are considered a CNS resident macrophages subpopulation [30,31]. Microglia provide metabolic support to neurons and cleaves cellular debris, i.e., organelle and apoptotic cells, via phagocytosis and autophagy [32]. Classically, microglia have been classified, depending on the surrounding environment, in either activated (pro-inflammatory, M1 phenotype) or non-activated (anti-inflammatory, M2) states [33], promoting opposite effects on neurons: M1 state induces neuronal dysfunction while M2 state stimulates neuroprotective mechanisms [34]. As an example, ALS patients carrying *SOD1* mutations displayed microglial activation [35,36]. Furthermore, activated microglial morphology has been detected in *post-mortem* ALS tissues, especially in *C9ORF72* mutation carriers, together with MN degeneration [37,38].

Some mutated *SOD1*, *FUS*, and *TDP-43* transgenic mouse models collect important ALS pathological hallmarks, such as motor alterations and premature mortality, as well as molecular ALS features, such as MN degeneration and glial cell activation [39,40,41]. Noteworthily, pathology-associated molecular mechanisms in TDP-43 are substantially different from SOD1. While TDP-43 inclusions are present in most ALS cases, including *TDP-43*, *FUS*, and *C9ORF72* mutation carriers, these aggregates do not appear in *SOD1* mutation carriers. Moreover, selective expression of *SOD1* and *TDP-43* specific mutations in mouse MNs is sufficient to induce neurodegeneration [42,43,44,45], but not in all cases [46], suggesting that other molecular mechanisms could be implicated in the pathogenesis. Surprisingly, overexpression of SOD1-G93A mutation in mouse microglia showed high expression of M2 phenotype at a presymptomatic stage and reduced M2 and increased M1 markers at the late stage of the disease [47]. In addition, mutated TDP-43 mouse models displayed upregulation of proinflammatory cytokines [48,49]. However, the selective expression of SOD1-G93A in microglia did not induce MN degeneration [50]. Finally, depletion of *C9ORF72* induced higher expression of proinflammatory cytokines, although without ALS-like pathology development [51,52]. Overall, there is relevant evidence that supports the important role of microglia in promoting MN degeneration. Further studies need to be conducted to better understand their implication in ALS pathology.

### 2.2. Astrocytes

Astrocytes have a leading role in the nutritional and energetic support of neurons in the CNS [53]. Yet, astrocytes share a common cellular lineage with neurons [54]. Like microglia, astrocytes are also commonly classified in A1 (neurotoxic) and A2 (neuroprotective) astrocytes. Indeed, reactive astroglia has been detected in ALS patients together with MN degeneration [55,56,57].

*SOD1* and *TDP-43* mutation carriers present astrogliosis [58,59], similar to *C9ORF72* poly (GA) repeat mouse models [60]. However, it remains unclear whether astroglial pathology or MN degeneration emerges first. Specific silencing of ALS SOD1-G85R mutation in astrocytes in transgenic mouse models resulted in milder phenotypes and delayed disease onset [61], suggesting an important role of those cells in ALS pathogenesis. Moreover, astrocyte-selective expression of TDP-43-M337V mutation caused astrogliosis, and importantly, the subsequent progressive neurodegeneration, supporting *non-cell autonomous* mechanisms’ theories [58]. In this line, *TDP-43* silencing in astrocytes triggered MN degeneration [62], while a similar approach in an in vitro design did not lead to MN degeneration [63].

On the other hand, neuroprotective mediators appear to be downregulated, whereas neurotoxic factors, such as LCN2 or CHI311, are upregulated in response to pathogenic *TDP-43* expression [58,64]. Furthermore, astrocytes carrying *C9ORF72* mutations showed higher levels of oxidative stress markers, inducing MN death when cultured with mutant astrocyte-conditioned media [65]. Finally, some metabolic pathways seem to be dysregulated in response to glutamatergic stimulus (or in co-culture with MNs); among them, lactate transport between astrocytes and MNs is unbalanced [66], indicating potentially pathological changes in cell metabolism.

The above-presented findings encourage us to convey the need to further explore the *non-cell autonomous* mechanisms as primary effectors of ALS pathogenesis. Besides microglia and astrocytes, oligodendrocytes are also important in ALS pathogenesis, playing a crucial role in axon myelinization. Indeed, oligodendrocyte alterations have been detected in both *post-mortem* tissue from ALS patients and in animal models [67,68,69]. Importantly, and supporting *non-cell autonomous* process relevance, oligodendrocyte degeneration has been detected in the spinal cord of *SOD1^G93A^* mice before the disease onset [70]. Moreover, TDP43-G298S fibroblast-derived oligodendrocytes led to MN degeneration when co-cultured via ROS, while *C9ORF72* iPS-derived oligodendrocytes promoted MN toxicity by ROS-independent pathways [71].

In turn, NMJ is a complex environment where numerous cell types interplay with factors, nutrients, and different functional molecular signals in a co-regulated system. Non-neuronal CNS cell types perform an important role regarding neuronal support and may contribute to MN degeneration under pathological conditions. Nevertheless, muscle represents one of the key players within the NMJ, not only as an effector organ but also as a releaser of molecules inducing selective molecular responses in all NMJ cell types.

### 2.3. Skeletal Muscle Cells

The skeletal muscle is considered as a solid low-turnover tissue that nevertheless maintains a robust regenerative capacity, with rapid re-establishment to full power occurring even after severe damage [72]. However, several lines of evidence suggest that muscle turnover is impaired in ALS. For instance, the capacity of myoblasts isolated from patients to form mature myotubes is decreased in vitro [73,74]. Likewise, myogenic defects are also reported in mouse myoblasts expressing ALS-linked mutations in *SOD1* or *VAPB* (Vesicle-Associated Membrane Protein-Associated Protein B/C) [74,75]. Another indication for defected muscle regeneration has been described from 66 patients of ALS or polyneuropathy, where evidence of some immature myofibers in muscle samples is suggested to arise from detached muscle progenitors that had fused to form new fibers in an “abortive” myogenesis attempt [76]. Notably, muscle atrophy has classically been considered secondary to denervation, as in ALS pathology, but activation of muscle progenitors has been confirmed in human biopsies/necropsies from symptomatic ALS patients [73,77].

Finally, recent evidence indicates that intense exercise can exacerbate neurodegenerative diseases, including ALS, possibly increasing ROS [78,79]. Interestingly, it has been latterly reported that circulating antioxidant levels are increased in “super-healing” *Murphy Roths Large* (MRL/MpJ) mice, and the deletion of *Sod1* in these animals impairs their myogenic potential [80]. This suggests that, indeed, ALS-linked gene mutations can modulate myogenesis and tissue repair independently of denervation.

## 3. The Skeletal Muscle in ALS Context

### 3.1. Specific Mouse Models Used in ALS Research

There is a large variety of genetic ALS mouse models to shed light on disease mechanisms, targeting different mutations in ALS-linked genes as *SOD1*, *TDP-43*, *C9ORF72*, or *FUS* [81]. The first and most broadly used ALS model is the *SOD1^G93A^* transgenic strain, covering a Gly^93^ →Ala (G93A) mutation in the second most common gene associated with ALS in humans [39]. Whole-body overexpressed human G93A missense mutation results in early disease onset and fast progression, cumulative MNs loss, and muscle atrophy [39] (Table 1). Due to its closeness to human symptoms, this model has been broadly studied and used for testing therapeutics intending to alleviate ALS symptoms [24]. Unfortunately, no effective treatment has been found thus far. Indeed, we know very little about the early pathogenic events in ALS, affecting different genes, sporadic in most cases, but with the same final clinical outcome. To elucidate the molecular pathology and consequences of ALS, different in vivo and in vitro models, mouse or human, are crucial for a better understanding of the disease.

As previously mentioned, ALS is now considered a *non-cell autonomous* disease with different cell types involved in the disease [11]. Indeed, MN degeneration occurs upon both intracellular and intercellular damages [82]. Moreover, in mutant *SOD1* models, downregulation of either SOD1-G37R mutation in motor and dorsal ganglion neurons, or SOD1-G85R mutation specifically in MNs and interneurons, delays disease onset but not its progression [83,84]. Furthermore, overexpression of SOD1-G37R mutation in all cell types except the MNs and the oligodendrocytes accelerates the onset of the disease [59]. Accordingly, whole body overexpression of human TDP-43-Q331K mutation except in MNs drives a significant degree of protection in NMJ at early stages but is unable to prevent age-dependent degeneration of axons and NMJ loss [85]. On the other hand, rescuing mutant SOD1-G37R or SOD1-G85R in the myeloid lineage (including microglia or macrophages) slowed down disease progression as well [83,84], suggesting a critical role of glial cells in the spreading of the disease from an initially damaged region [82]. Supporting these data, Wang and collaborators demonstrated that deletion of mutant SOD1-G85R in all cell types from a *G85R^floxed^* mouse model slightly ameliorates the disease by delaying onset and lengthening duration [86]. Finally, specific modulation of these genes in the skeletal muscle locates this tissue as the main player in ALS disease [18] (Table 2), which in some cases display an ALS-like phenotype [18,87].

As our goal in this review is to highlight the relevance of the skeletal muscle in ALS, this section discusses how modulation of ALS-associated genes in mouse models specifically in the skeletal muscle, results in an ALS-like phenotype, coursing with neurodegeneration and atrophy [18], whereas modulation in other cell-types (MN, astrocytes, glia) does not [42,43,95,96]. In neurons, specific overexpression of either human WT *SOD1* or mutant SOD1-G37R forms does not cause motor impairments, the animals remaining healthy up to 1.5 years of age [42]. On the other hand, restricted expression of mouse Sod1-G86R (SOD1-G85R in humans) in astrocytes results in astrocytosis with no MN degeneration, as in many of the studied mutations involving *SOD1* or *TDP-43* [96]. Finally, Lino and collaborators demonstrated that specific accumulation of *SOD1* mutations specifically in MNs does not cause MN pathology/disease [43]. These data confirmed that specific modulation of SOD1 in neural cells was not sufficient to cause MN degeneration in vivo, suggesting that ALS pathogenesis may involve non-neuronal cells.

Aiming to understand ALS pathogenesis, researchers have knocked down different ALS-linked genes intending to understand their corresponding functions. However, there is no evidence of such gene deletion in ALS patients. With these approaches, knocking down *Sod1* in neurons and the skeletal muscle did not result in muscle atrophy [88,95], but that deletion in the skeletal muscle increased weakness and the presence of regenerative fibers. Another model with *TDP-43* overexpressed specifically in the skeletal muscle reduced body weight due to an upregulation of cellular stress such as the unfolded protein response (UPR) system [93]. However, an increase in the expression of *TDP-43* has not been described in ALS patients, only in other diseases such as Inclusion Body Myositis (IBM). These experiments underlined the importance of the specific gene function in the targeted tissue. However, some mutations of loss- or gain-of-function are under consideration, as some features of the disease can be due to either an incorrect/absent functionality of the mutated genes or to an increase in toxicity [7,24,81,97].

As previously mentioned, ALS patients display a strong muscle phenotype, including progressive atrophy and wasting [98], sharing some mechanisms with other neuromuscular diseases including age-induced sarcopenia [99]. Actually, many of the alterations that take place in the CNS, and especially in the MNs, are replicated in the skeletal muscle [18,87] (Table 1). To unveil the specificity of ALS mutations exclusively in the skeletal muscle, many mouse models have been generated (Table 2), with stronger pathological outcomes than those generated by overexpression of similar mutations solely in the CNS. Indeed, some of these models display an ALS-like phenotype in the skeletal muscle, but also in the NMJ and in the MNs [17,18]. In this case, the overexpression of *SOD1* and related mutations in the skeletal muscle induces muscle atrophy, NMJ dismantlement, axonopathy, and MN loss. Moreover, in the *MLC/SOD1^G93A^* mouse model with SOD1-G93A mutation expressed specifically in the skeletal muscle, Dobrowolny and colleagues described modulation of different microRNAs and the mRNA transcription pattern associated with the myelination process in the spinal cord of these mutants [90]. Finally, in the *SOD1^G93A^* model, overexpression of *Igf-1* (insulin-like growth factor 1) in the skeletal muscle not only rescues the muscle phenotype but also gains NMJs stabilization, enhances MN survival, and more importantly, delays the onset and progression of the disease [89]. This study concludes that the skeletal muscle might be a primary target in ALS, suggesting that muscle fibers-derived factors are needed for neuron survival.

### 3.2. Muscle Atrophy in Disease

The loss of MNs and atrophy of the associated muscle are common features of MN diseases, making such disorders severely debilitating and usually fatal. Indeed, the term “amyotrophic” in ALS refers to the muscle atrophy, weakness, and fasciculations that reflect disease of lower MNs [100]. Certainly, muscle weakness is the most apparent symptom in ALS patients. Whereas the damage of the upper MNs loss is related to spasticity, hyper-excitability, and the appearance of pathological reflexes, the damage of the lower MNs death leads to muscle weakness and atrophy, which are followed by progressive paralysis [101,102,103,104,105]. Similarly, a progressive and generalized degenerative loss of muscle mass, strength, and function occur in physiological aging, known as sarcopenia [106,107,108]. Like in ALS, fast muscle fibers atrophy preferentially than slow myofibers [109,110]. In both ALS patients and rodent models, there is the selective vulnerability of fast-fatigable MUs, and type IIB/X muscle fibers are the most susceptible and earliest lost, while slow type synapses are more resistant and affected last [111,112,113,114,115,116,117]. Studies conducted on murine *SOD1^G93A^* muscles demonstrated that the preferential denervation of type IIB fibers progressively results in an increase of type I and IIA myofibers due to a collateral sprouting of the axons coming from the surviving slow-twitch MUs [113,117,118,119,120]. It remains unanswered whether such loss in muscle mass results from the motor unit (MU) loss [109,121], with atrophy of muscle fibers occurring subsequently [109], or whether it is due to loss of proteostasis and/or related to muscle aging [20]. However, in an animal model of sarcopenia, abnormal NMJs and MN death were attributed to muscle trophic support to neurons being defective [122]. This points out that MNs fitness depends on trophic factors provided by muscle. Denervated fibers that atrophy, and continuous cycles of denervation and reinnervation by decreased-force MNs, cause the characteristic muscle weakness in ALS. Related to this observation, Di Pietro and collaborators found that the skeletal muscle of ALS patients with longer survival periods expressed higher levels of microRNAs involved in the regulation of slow-to-fast fiber type switch profile [123].

On the other hand, it is still unclear whether muscle atrophy in ALS results solely from the loss of muscle activity due to denervation or whether intrinsic pathologic mechanisms within the muscle could induce such wasting. Since the initial description of ALS [124], muscle atrophy has classically been considered a secondary process following MN loss and denervation. Indeed, electrophysiologic traits found in ALS muscle [125,126], as well as histopathologic changes such as the selective vulnerability of fibers and fiber type grouping [127], coincide with those of denervation-induced atrophy. However, functional studies in ALS patients point towards an early and active role of the skeletal muscle in the development of ALS [128]. Other pathologies distinct from those of denervation-induced atrophy have been described in ALS muscle, including myopathic features and fiber necrosis and inflammation [129,130,131]. This suggests that the observed muscle atrophy in ALS is not just a consequence of denervation but that primary myogenic defects could be involved in the etiopathogenesis of the disease. Muscle RING finger 1 (*MURF1*) and muscle atrophy F-box (atrogin-1/*MAFBX*), two muscle-specific E3 ubiquitin ligases from the ubiquitin-proteasome system (UPS), are well-established markers of muscle atrophy by increasing their expression in atrophy conditions [132,133,134]. Leger and colleagues found that the expression of atrogin-1 was increased in both mutant *SOD1^G93A^* mice and human ALS samples, while no changes in expression were reported for MURF1 [135]. Consistent with these findings, a significant increase in atrogin-1 mRNA and protein content associated with reduced AKT activity was reported in biceps and vastus lateralis muscle in a cohort of ALS patients [135]. Similarly, the low expression of AKT protein was found to be correlated with unfavorable prognosis and overall survival of ALS patients [136]. The IGF-1/PI3K/AKT signaling pathway is known to stimulate muscle hypertrophy while its inhibition results in muscle atrophy [132,137,138]. Several components of this system, including IGF-1, are decreased in the skeletal muscle of sALS patients, while the IGF-1 receptor β subunit (*IGF-1Rβ*) was significantly increased, and the expression of activated AKT was downregulated in human ALS skeletal muscle [135,139]. Studies conducted in the *MLC/SOD1^G93A^* mouse model (Table 2) showed that the PI3K/AKT pathway initiates the SOD1-mediated atrophy by suppressing protein synthesis and inducing *FOXO3* mediated expression of atrogin-1 and MURF1 [17]. Furthermore, Dobrowolny and collaborators showed that this atrophy is independent of MN degeneration and the activation of apoptotic markers [140]. Actually, the activation of caspases mediates apoptosis later upon muscle denervation at a late stage of disease, which exacerbates the atrophic phenotype and causes a shift in the fiber-type composition [140].

Additionally, muscle atrophy generally occurs due to an imbalance in proteostasis, where protein degradation exceeds protein synthesis and results in loss of contractile proteins and shrinkage of myofibers, which ultimately lead to loss of muscle mass and muscle weakness [141]. At the molecular level, muscle fiber atrophy can be attributed to different signaling pathways, which are relevant to the abnormality of protein degradation. Indeed, protein aggregates with TDP-43 [142,143], neurofilament [144], FUS [145], or SOD1 [146], which are detected in the vast majority of ALS patients, can appear in the cytoplasm of neurons [147] and within the skeletal muscle [148,149], suggesting an imbalance between protein synthesis and degradation pathways. In fact, there is solid evidence that defects in the two major protein clearance pathways, the UPS and the autophagy, mediate such dysregulated protein homeostasis and can be central components of the disease mechanisms in ALS. The identification of mutations in genes encoding ubiquilin 2 (*UBQLN2*) [150] and *VCP* [151], two proteins involved in protein clearance via the ubiquitin-proteasome pathway [152], were the first signs that proposed that the UPS could be dysregulated in ALS. Mutations in other genes involved in such a system were identified later: optineurin (*OPTN*) [153], *SQSTM1/P62* [154], *VABP* [155], *C9ORF72* [147,156], and cyclin F (*CCNF*) [157]. Besides, ubiquitin-positive inclusions, for instance, in patients carrying a pathological hexanucleotide-repeat-expansion in *C9ORF72* [158], have been detected in both familial and sporadic ALS patients in *post-mortem* neuronal and muscular tissues [159]. Other ALS-related proteins such as SOD1 [160], FUS [145], UBQLN2 [161], and C9ORF72-derived dipeptide repeat (DPR) proteins [162,163], have been found within toxic aggregates which are positive for several proteasome components [164], while nonmutated forms of *TDP-43*, *OPTN*, and *UBQLN2* have been observed in such ubiquitinated inclusions, further aggravating the disrupted cellular homeostasis in ALS. Strikingly, these toxic aggregates can be released and disseminate, amplifying existing proteostatic imbalances, and propagating the pathology in vulnerable cells, such as MNs [165].

Besides, the NFκB transcription factor family activates the UPS via upstream signaling of molecules such as cytokines or ROS [166,167]. Halter and colleagues provided further evidence supporting that the loss of muscle homeostasis occurs prior to denervation and the initiation of motor symptoms [168]. They found that the accumulation of ROS produced by muscular mutant SOD1 was coincident with the increase in the expression of Ras-related associated with diabetes (Rad), an inhibitor of voltage-gated calcium channels previously found to be upregulated in muscle [169], prior to the manifestation of motor symptoms. This study revealed the implication of oxidative stress in the modulation of Rad expression and concluded that there are pathological modifications related to the presence of oxidative stress within the muscle fibers that do not derive from MN injury [168]. Another main proteolytic system that controls atrophy, the autophagy-lysosome system, also seems to be involved in intrinsic muscle defects in ALS. The transcript levels of genes such as *Map1lc3* (LC3 protein), *Bnip3*, and cathepsin L have been reported to be upregulated in muscles from *MLC/SOD1^G93A^* mice, and are suggested to contribute to the decrease of myofiber size [17,140]. Besides, *C9ORF72* mutations can interfere with the autophagy pathway at several levels (reviewed by [97]), and in particular, DPR proteins have been found not to be limited to neurons but also been detected in Sertoli cells [170], ependymal cells [171], and in the skeletal muscle of a zebrafish *C9orf72* model [172], *Drosophila C9orf72* model [173], and ALS patients [162]. Effectively, the latter authors previously demonstrated the presence of phosphorylated TDP-43-positive (FUS-negative) aggregates in myofibers of ALS patients, which were also positive for the autophagy pathway SQSTM1/p62, suggesting the possible implication of endogenous autophagic mechanisms in ALS muscles [174]. Together, these pathway alterations reflect that muscle-intrinsic atrophy mechanisms contribute to decreased myofiber size in ALS.

### 3.3. Perturbations of Energy Metabolism in ALS Muscle

#### 3.3.1. Muscle Exercising and The Risk of ALS

Although ALS shares several pathological traits with other neurodegenerative conditions, such as proteinopathic inclusions of TDP-43 or ubiquitin, in contrast to the rest of neurological conditions, exercise might be a risk factor for the development of ALS. Various prospective cohort and case-control studies support the association between frequent physical activity and the risk of developing ALS [78,175,176,177], and other longitudinal and cross-sectional observational studies have found over-representation of athletes and professional soccer players among populations of ALS patients [178,179,180]. However, this relationship has not been replicated in all studies [181], and, therefore, it remains controversial. Actually, a recent meta-analysis study has stated the lack of sufficient evidence to draw a firm conclusion on the relationship between physical activity and ALS, highlighting limitations in previous studies related to the heterogeneous or inadequate classification of physical activity [182]. Thus, not every level of exercising seems to affect the risk of ALS equally. Those studies that have classified exercise into different intensity categories have indeed led to the conclusion that strenuous and frequent physical activity, but not moderate, does associate with increased risk for developing ALS [78]. However, once the disease is developed, exercise exerts benefits on patients as it improves their functional scores [183]. Besides, a study using big data and Mendelian randomization approach has found an association between genetic liability to strenuous sport exercise and ALS risk [184], supporting the idea of a vulnerability to sALS in the context of high levels of physical activity. This indicates that forced and excessive usage of skeletal muscle resources during early and mid-life might be a pathogenic factor in genetically predisposed individuals, but the underlying mechanisms that account for this association are still far from being delineated.

Exercise-induced muscle damage and microinjuries ascribed to mechanical disruption of the fiber and subsequent inflammatory processes and changes in excitation-contraction coupling within the muscle, although not yet studied, might provide a compelling explanation for this association. In this sense, one of the most studied leisure time factors associated with the risk of developing ALS (and FTD) is head trauma in collision sports [185], which has been generally attributed to traumatic brain injury and neurovascular lesions. However, fragmentary unpublished clinic-based observations have perceived a sort of causal correlation between the site of muscle damage (either surgery-induced or accidental) before ALS diagnosis and the region of symptoms’ onset. Indeed, there is reported evidence showing that cases with repeated head injuries are more likely to have an early and bulbar onset of ALS [186], suggesting a relationship between cranial/neck muscle injury and the onset of bulbar ALS symptoms. Thus, further studies should interrogate the hypothesis that focal muscle damage and subsequent inflammatory processes, either induced by physical activity or not, can precipitate the onset of disease. Beyond this hypothesis, other potential mechanisms underlying the relationship between frequent, strenuous exercise and ALS risk can be inferred from the current empirical evidence. Being the skeletal muscle is a very metabolically active tissue, one point that deserves much attention is how homeostasis of energy metabolism in muscle can impinge on MNs and ALS pathogenesis, which will be reviewed and discussed next.

#### 3.3.2. Alterations of Muscle Metabolism as Prodromal Features of ALS

We have previously acknowledged that MNs supplying type IIB muscle fibers are the most vulnerable to ALS degeneration [187,188]. Interestingly, these fibers are responsible for anaerobic burst activity, which is the type of metabolic pathway more frequently required in strenuous sport exercising. In contrast, MNs supplying type I aerobic fibers are less vulnerable; a case in point is the observation that MNs innervating extraocular muscles are relatively resistant to ALS neurodegeneration [187]. Extraocular fibers are cells with the highest numbers of mitochondria in the body, which rely almost exclusively on the energy obtained in the mitochondrial respiratory chain [189], and apparently the most susceptible cells to mutations in mitochondrial DNA. As aerobic (extraocular) muscle fibers are particularly affected in mitochondrial encephalomyopathies caused by mutations of mitochondrial DNA (i.e., Kearns–Sayre syndrome, whose main clinical sign is progressive external ophthalmoplegia), it is plausible to infer MNs of the anaerobic motor units being more vulnerable to defects in muscle glycolytic metabolism. Considering that glycolysis produces much less energy than oxidative respiration, the question that naturally arises is as to what extent repetitive cycles of muscle energy shortage over the years may contribute to ALS MN degeneration. Frankly, a bulk of experimental work made during the last decade has revealed the peripheral perturbations of energy metabolism, which are linked in some way to ALS pathology [7].

The first evidence of energy dyshomeostasis in ALS was collected in 1996, when whole body hypermetabolism (in terms of increased resting energy expenditure) was described in non-ventilated ALS patients. Initially, this hypermetabolic phenotype appeared paradoxical because ALS patients often experience significant reductions in fat-free mass (skeletal muscle), which is the principal determinant of resting energy expenditure. Hence, hypermetabolism was attributed to the increased respiratory work needed from weakened muscles to maintain appropriate gas exchange [190]. Further studies confirmed that the relationship between hypermetabolism and ALS was independent of the forced vital capacity (a lung function test) or any other known hypermetabolic determinants, such as hyperthyroidism, infection, or smoking [191,192]. Finally, the latest studies have concluded that hypermetabolism is not present in all cases but associated with a worse prognosis in those where this condition is present [193,194], which is actually estimated in more than 50% of patients [191,194]. Hypermetabolism is a phenomenon commonly defined as an adaptive response to various types of injuries, including sepsis, trauma, or severe burns, to cope with the inefficiency with which energy is utilized in the area of injury or infection. Consequently, carbohydrate, protein, and fat stores are generally mobilized to satisfy these prolonged increased energy demands, thus resulting in hyperglycemia and whole-body catabolism [195]. Specifically, the hypermetabolic response promotes lipolysis of the white adipose tissue and proteolysis from skeletal muscle to increase the release of free fatty acids (FFA) and glycerol, and amino acids, ultimately resulting in significant elevations in resting energy expenditure. Unfortunately, high levels of FFA may overwhelm mitochondria’s ability to metabolize the substrate, leading in turn to increased fat deposition in the organs. According to this response, an alternative proof of hypermetabolic signs in ALS comes from the evidence reported by two independent studies that indeed observed accumulation of fat deposits in livers of a quite big proportion of ALS patients [196,197]. Moreover, dyslipidemia in patients with ALS, indicated as either increased levels of triglycerides or LDL in the blood, has been repeatedly described, although with controversial data regarding its association with disease outcomes [196,198].

Hypermetabolism promotes proteolysis and consequently induces skeletal muscle wasting and atrophy, a phenomenon that can be masked in ALS patients due to an overlapping muscle wasting induced by denervation. What deserves special attention in the present review are the primary metabolic changes that take place intrinsically in the skeletal muscle and eventually can initiate the hypermetabolic response; and how they can impinge on MN neurodegeneration. Transgenic animals have shed some light on this issue, developing dramatic muscle atrophy prior to spinal MN loss [18,87], which unquestionably indicates the presence of primary and intrinsic muscle wasting mechanisms in ALS. Additionally, it has been observed that the restricted expression of mutant *SOD1* in the skeletal muscle induces a fast-to-slow switch in muscle fiber composition before the onset of ALS symptoms and MN loss [199]. This switch may be indicative of poor glycolytic performance in muscles, and thus, glucose utilization was found decreased in these mice in exchange for a preference for lipids [199], which responded to an upregulation of pyruvate dehydrogenase kinase 4 [91]. Indeed, another study confirmed glucose intolerance accompanied by an increase in fatty acid oxidation in the skeletal muscle preceding hypermetabolism in presymptomatic *SOD1^G93A^* mice. The use of Ranolazine, a drug that improves glucose utilization as fuel, partially abrogated the hypermetabolic phenotype and induced a temporary recovery of ALS traits in this mouse model [200]. Together, the evidence achieved in experimental rodents that mimic SOD1-related ALS support the hypermetabolic traits observed in patients and highlight the primary role of metabolic defects that occur in the skeletal muscle before disease symptoms and independently of MN degeneration. This indicates that the skeletal muscle is likely an important therapeutic target in ALS.

## 4. Pathological Spreading into Neurons: Mechanisms

As mentioned above, overexpression of mutant *SOD1* in the skeletal muscle affects MN viability, suggesting that the physical communication between skeletal muscle and nerve influences neuronal survival, axonal growth, and maintenance of synaptic connections in ALS [11,17]. Indeed, analysis of the *MLC/SOD1^G93A^* muscle-specific mouse model determined that ion channel function was impaired, leading subsequently to muscle hyperexcitability [201]. These findings emphasize the important involvement of the skeletal muscle in ALS, and at the same time, suggest that the oxidative stress generated by the selective accumulation of mutant SOD1 in the muscle may induce alterations in ionic conductance. These alterations may then initiate distal hyperexcitability, ultimately leading to MN death through the loss of NMJ’s integrity. Thus, these data suggest that interactions between the different cell populations, muscle, NMJ components, and MNs are affected. Therefore, if muscle participates actively in the neurodegeneration process, there must be plausible mechanisms that may explain the spreading of the toxicity to neurons. Although this issue is not fully resolved yet, several works have shed light on the mechanistic connection to explain the retrograde spreading of ALS disease hallmarks.

Muscle is an extremely robust tissue, subjected to strain forces much greater than for any other tissue. This mechanical activity frequently leads to damage, thus that new myofibers must replace the damaged ones [72]. Additionally, signaling must be critical to coordinate the different agents required for new NMJs formation [202,203]. Interestingly, exercise, as main source of muscular micro-lesions, regulates the release of different secreted factors that might be relevant for ALS pathogenesis [204]. On the other hand, cross talk between muscle-MNs through the NMJ will be essential for tissue preservation and regeneration. As so, after an insult, satellite cells (SCs), the bona fide stem cells required for muscle regeneration, can exit their dormant stage entering an alerted status [205]. This alert is transmitted along the body by secretion of different cytokines/factors not only to other SCs in distant muscles but also to the immune system and endothelium [205,206]. Therefore, signals coming from new forming myofibers and SCs implied in the process could be crucial in the context of preventing motor loss. This revolutionary data implies beneficial consequences in the understanding of the disease, especially when it has just been described as a SC secretory function that is essential for NMJ preservation [207].

Muscle possesses three different ways to communicate with the surrounding cells: (a) cell-cell communication [208], (b) secretion of paracrine substances (myokines, cytokines and other factors including microRNAs) [204], and (c) release of extracellular vesicles (EVs) and exosomes [209]. All mechanisms can potentially influence the status of neurons. Both muscle and neurons possess transmembrane proteins that might interact to regulate axonal growth and NMJ integrity [210]. The neurite outgrowth inhibitor (NOGO), encoded by the reticulon 4 gene (*RTN4*), is a well-established mediator of such effects by destabilizing motor nerve terminals and promoting denervation [211]. ALS patients show high levels of NOGO-A isoform in skeletal muscle biopsies [208,212], which led to the use of NOGO inhibitors in ALS clinical trials, but with no success [213]. Indeed, NOGO activity has been shown to be critical for muscle regeneration [214]. Thus, despite the neurogenic effects of NOGO inhibition, this approach might impair muscular functionality, which may explain the therapeutic failure.

On the other hand, the skeletal muscle also releases different molecules such as interleukin-6 (IL-6) and -8 (IL-8) or brain-derived neurotrophic factor (BDNF) [215,216,217]. IL-6 is a modulator of the inflammatory response [218], which additionally has been shown to modulate metabolism contributing to insulin resistance [219,220]. In skeletal muscle, IL-6 inhibits insulin-mediated glucose uptake [204,219,220]. In neurons, the IL-6 pathway has been successfully used to elicit axonal regeneration after surgical excision after both optic nerve and spinal cord injuries [221,222]. However, the role of IL-6 in the low MN is unknown. ALS meta-analysis has shown elevated IL-6 in blood samples [223], likely evidencing a compensatory role. IL-6 was also elevated in the spinal cord of *SOD1^G93A^* mutant mice [224]. However, knocking out *Il-6* in this model did not change the course of the pathologic markers. These studies indicate possible compensatory mechanisms in ALS mediated by IL-6 to stimulate nerve growth, although more studies are required to evaluate the source of elevated IL-6 levels. Conversely, BDNF is a very well-described neurotrophic agent that has been implicated in several neuronal diseases, including ALS [225]. BDNF is also synthesized by the skeletal muscle and stimulates neuronal growth when overexpressed in muscle tissue [226]. Interestingly, BDNF seems to be expressed in differentiating precursors rather than in mature myofibers [227], indicating that BDNF signaling participates in myogenic processes. Unfortunately, the use of BDNF as a therapeutic target in ALS has failed in preclinical and clinical trials [228]. Several other myokines have been shown to be released by muscle [204], but it is much less clear whether they could elicit any kind of influence in MNs. Further studies might shed light on the involvement of these other factors in the future.

In addition, the skeletal muscle has been shown to be very active in the production of exosomes and EVs. These exosomes release their content directly into the cytoplasm of the target cell. Additionally, the vesicular content is much more diverse, including not only proteins but also RNA mediators [202]. Interestingly, EVs produced in muscle differentiating progenitors are critical for muscle repair [202,203,209]. Given that, ALS-relevant proteins such as TDP-43 are required to translocate from the nucleus into the cytoplasm to allow successful myogenesis [94]. Thus, ALS aberrant TDP-43 in muscle cells might induce the release of toxic exosomic content, which would spread the pathology to the neuronal tissue. Notably, excellent preliminary work has shown that ALS patient-derived myotubes increase EVs production with changes in their cargo which becomes neurotoxic [229]. Furthermore, this cargo is enriched in molecules involved in RNA processing. Among them, FUS demonstrates to possess toxic properties when applied to healthy iPSC-derived MNs [229]. Strikingly, to our knowledge, this is the first time that a toxic effect directly from the muscle targets MNs death. Supporting these data, in vitro generated neuro-myotube co-cultures carrying ALS relevant mutant versions of FUS in the myotubes were sufficient to alter NMJ formation [230]. Therefore, it is plausible to accept that EVs are responsible for a pathological spreading of muscle alterations to MNs, through the NMJ, following the “dying back” hypothesis. These outstanding results imply the development of new therapeutic approaches targeting either EVs or their cargo.

Finally, several studies have shown that the pathogenicity related to the GGGGCC hexanucleotide-repeat-expansion within the first intron of the *C9ORF72* gene, which is the most frequent mutation found in patients of ALS and also a major cause of frontotemporal dementia (FTD) and the ALS/FTD overlap syndrome [149,231,232], might be related to the production of arginine-rich cell-penetrating peptides (CPPs). GGGGCC expansions are translated into several DPRs in both sense (poly-GP, poly-GA, poly-GR) and anti-sense (poly-GP, poly-PR, poly-PA) directions, and such products have been detected in *post-mortem* tissue [170,233,234,235,236]. The toxicity driven by arginine-containing DPRs has been demonstrated in both cell cultures [163,237,238] and in animal models [60,163,239,240,241,242,243,244]. Recently, Lafarga and colleagues suggested that the presence of poly-PRs produces a generalized displacement of RNA- and DNA-binding proteins from chromatin and mRNA, impairing processes such as RNA transcription, translation, splicing, and degradation, or DNA replication and repair [9]. In fact, alterations in all these processes have been linked to ALS/FTD [245]. Since DPR pathology is not restricted to CNS but also present in skeletal muscle of at least ALS patients carrying the *C9ORF72* repeat expansion [162], CPPs represent plausible mediators for spreading toxicity from the muscle to the MN.

### Axonopathy—The Axonal Degeneration

Axonal degeneration is one of the most reliable pathologic factors associated with ALS, and the accumulation of cytoskeletal components in neurofilaments with TDP-43 is one of the classical hallmarks of the illness [246]. These TDP-43 aggregates have also been found in neuronal, glial, or Schwann cells and in the skeletal muscle [174,247]. The vast majority of genes associated with ALS are not exclusively expressed in neurons and, the alterations associated with them, i.e., RNA metabolism or protein degradation, might also occur in other tissues, including the skeletal muscle (Table 1). Nevertheless, some ALS-associated genes are predominantly expressed in neurons, such as *TUBA4*A, *PRPH*, or *OPTN* [248]. The existence of these genes might argue against a central role of the skeletal muscle in ALS pathogenesis. However, these neuron-specific genes turn out to be mainly related to vesicular trafficking and cytoskeletal dynamics [249]. As this function must be key for recognizing signaling coming from the skeletal muscle, these genes might work as vulnerable factors to aberrant signals coming from the skeletal muscle, which would progress retrogradely, leading to the spreading of the pathology.

The great extension from the soma to the pre-synaptic terminals in the MN requires that the somatic region and the distal axonal region exchange materials, thus that the soma responds to demands in the axonal region with the appropriate changes in gene expression. This communication is mediated by the axonal transport using the axonal microtubules as a scaffold to transport vesicles faster than passive diffusion. Axonal transport requires certain proteins to take place successfully. Several kinesins participate in the anterograde transport, pointing towards a potential specialization of different protein family members for different EVs cargo. Amongst them, kinesin-5A (KIF5A) seems to participate in mitochondrial transport [250], and its genetic ablation disrupts axonal trafficking [251]. Interestingly, mutations in KIF5A C-terminal domain have been associated with the development of ALS [252]. On the other hand, dynein, responsible for retrograde transport, requires the modulation of dynactin. Dynactin interacts with dynein and the microtubule through the p150Glued subunit, encoded by the gene *DCTN1* [253]. Moreover, DCTN1 is also involved in the transport of endosomes and lysosomes [254], and it is expressed in many cell types, including the skeletal muscle. Mutations such as G59S have been linked to non-ALS MN disease [255]. This mutation is located in the microtubule-binding domain of the protein and affects axonal transport without altering cell transport functions [256]. However, misallocation of lysosomes after DCTN1-G59S overexpression in vitro has been described [256]. Conversely, little is known about mechanisms mediating other ALS-related mutations (G95R, T1249I, M571T or R785W) [253,257] and how they could contribute to ALS pathology.

Axonal transport requires the integrity of the cytoskeleton. Several genes coding for cytoskeletal components has been linked to ALS [7]. Peripherin (PRPH), a structural protein predominantly expressed in neurons, is required for the intermediate filament ensemble [258]. It is upregulated during axonal growth and regeneration, indicating that it might be recruited during nerve regeneration processes [259,260]. Therefore, it is very likely that *PRPH*, also expressed in muscle, participates in the generation of new NMJs, favoring axonal guidance. Mutations in another component of the intermediate filaments, the neurofilament heavy chain (*NFH*), have also been linked to ALS [261]. Interestingly, neurofilaments and peripherins, are major components of the neuronal inclusions in affected ALS MNs [262]. These inclusions might indicate a profuse dismantling of the axonal cytoskeleton that overwhelms the proteolytic machinery. Finally, microtubules might also participate in the physiopathology of ALS. Several mutations in the tubulin beta-4A gene (*TUBA4A*) destabilize the microtubules impairing re-polymerization [263], likely contributing to the axonal degeneration of the MN.

Interestingly, ALS-related mutations in *SOD1*, *TDP-43*, or *FUS* trigger distal axonopathy. For instance, SOD1-G93A mutation seems to affect both anterograde and retrograde transport in presymptomatic murine models [264,265]. However, the mechanistic explanation for this fact remains elusive. Given that the specific muscle overexpression of SOD1-G93A recapitulates most ALS alterations [17,18], skeletal muscle SOD1 could play a crucial role in the distal axonopathy associated with ALS. Moreover, plausible mechanisms have been proposed regarding ALS-related gene mutations involved in RNA processing proteins as TDP-43 or FUS. Indeed, both proteins regulate *HDAC6* expression [266], whose deacetylase activity regulates microtubule stability [267,268]. Furthermore, FUS also regulates the expression of anterograde transport proteins KIF5C, KIF1B, and KIF3A [268,269] and microtubule-associated proteins such as Tau [270]. The presence of FUS or other ALS-related proteins within muscle EVs [229] suggest a central role in the pathogenic spreading of the disease from the skeletal muscle to the neurons. Even if distal axonopathy has been classically studied as a mechanism of *cell autonomous* toxicity in neurons, the aforementioned factors might implicate other toxicity mechanisms originated from the skeletal muscle in a *non-cell autonomous* manner. Future studies regarding muscle to neuron communication might elucidate how muscular alterations affect axonal transport in the context of ALS.

Finally, it is important to note that pathological alterations, including distal axonopathy could primarily appear in the upper MN as well, spreading to the lower MN. However, although this fact is widely accepted (dying forward mechanism), where lower MN will be presumably affected, the spreading of ALS pathology from the lower MN in a retrograde way to the upper MN (“dying back” mechanism) still remains unexplored. To explain the “dying back” phenomenon, several mechanisms have been proposed such as the prion-like propagation to presynaptic neurons and other adjacent cells [271]. Indeed, it has already been shown that pathological TDP-43 aggregates can be transferred from cell-to-cell in a similar way to those of prion-like proteins [272]. Moreover, the prion-like mechanism has been suggested to be common to other neurodegenerative diseases, based on different neuropathological similarities, including neuronal loss, accumulation of protein aggregates, gliosis, and brain atrophy [273]. According to this theory, TDP-43, FUS, or SOD1 could be included among the proteins capable of forming these aggregates in ALS [274,275,276,277,278]. An abnormal folding process of these proteins could induce their accumulation in the vulnerable cell and the subsequent propagation throughout the CNS [279]. Many mutations in *TDP-43*, *FUS*, or genes coding for other ribonucleoproteins related to ALS are found in regions known as prion-like domains, prone to cytoplasmic aggregation [280]. In addition, there are local environmental causes capable of triggering an abnormal folding, including persistent oxidative and metabolic stress [281]. Once formed, the aggregates of these ribonucleoproteins could consecutively induce the aggregation of the soluble fraction of other proteins by transmitting backward from the muscle to the lower MN, and then to the upper MN. Thus, the question of which MN, upper or lower, drives the global degeneration is still controversial. Indeed, both pictures are not fully mutually exclusive and likely could occur together [282].

## 5. The Neuromuscular Junction in ALS

The NMJ is the specialized synapse that connects the cholinergic MN ending with muscle fibers, essentially for muscle contraction. Each muscle fiber has a specialized post-synaptic area in which nicotinic AchRs (nAchR) are clustered, forming a characteristic Pretzel-like structure that will interact with the motor nerve ending. However, besides the MNs and muscle fibers, Terminal Schwann Cells (TSC) and the recently discovered kranocytes are also components of the NMJ [283,284]. TSCs are close to the nerve terminal and are essential to detect and modulate synaptic communication, regulate NMJ stability, and oversee NMJ repair [285,286]. The loss of MNs causes the denervation of entire motor units (MU) that become re-innervated by the expansion of other functional MUs. TSCs extend processes for MN axon guidance from the innervated to the previously denervated synaptic areas, giving them an essential role in NMJ development and regeneration [287]. TSCs can also adopt a phagocytic phenotype on denervated NMJs in order to phagocytose synaptic debris, which is also necessary for reinnervation [288]. Regarding kranocytes, these cells cap the NMJ above TSCs and are shown to respond to NMJ insults [289]. Therefore, it is evident that NMJs are active structures, where proper functioning of all components is necessary to preserve its integrity.

NMJ dismantling might play a crucial role in the onset of ALS through the ‘‘dying back” axonopathy [14,15,290,291]. Indeed, growing evidence describes MN and other CNS cell-types (i.e., glia or astrocytes), muscle, and TSC abnormalities in ALS pathophysiology [99]. Both patients and different in vivo ALS models have shown altered functional and morphological behavior of the different NMJ components, leading to NMJ destabilization and dismantlement. Furthermore, it has recently been demonstrated that NMJ denervation in ALS is a complex and dynamic process of continuous denervation and innervation rather than a manifestation of global MN degeneration [292]. Certainly, changes in NMJ transmission seem to be initiated long before symptoms onset, which makes it an important possible target for future therapies [293,294,295,296].

### 5.1. Pre-Synaptic Affectation

MN degeneration has been studied for decades in ALS pathology. However, distant in the axon terminal before MN death happens, several events contribute to a non-regenerative environment that leads to NMJ denervation, which is a classical hallmark of ALS [99]. NMJ denervation is a highly dynamic process where imbalanced denervation and reinnervation processes finally lead to unsuccessful reinnervation and consequent nerve-ending retraction [292]. Studies in *SOD1^G37R^* mice have demonstrated that MUs are dismantled asynchronously, starting slowly in a local branch-specific manner and then followed by a sudden global axonal degeneration [292]. This suggests that MN degeneration is the reflection of an exhausted system that is not capable of functional reinnervation. Besides, the probability of denervation also depends on NMJ location: NMJs on distal axon branches are more susceptible for NMJ loss than proximal ones, as proximal branches preserve the sprouting capability to extend toward NMJs from other MUs [292]. Tallon and collaborators have also observed that axonal degeneration was a length-dependent process, being the longest axons innervating the caudal region more vulnerable, reinforcing the “dying back” phenomenon [15].

Additionally, changes in MU-specific synaptic properties were observed in ALS animal models even before NMJ morphological alterations and MN degeneration [294,295]. Fast fatigable (FF) MUs showed decreased neurotransmitter release in presymptomatic *SOD1^G37R^* mutant mice, while increased synaptic vesicular content was observed in slow (S) and Fatigue Resistant (FR) MUs [295]. These packaged vesicles (quantum) changes persisted until disease onset, opposite to their intrinsic synaptic properties in a homeostatic environment with increased content in FF MUs. Quantum release and synaptic transmission attenuation were also observed in *TDP-43^Q331K^* and *FUS* mutant mice, reinforcing the role of RNA-binding proteins in ALS pathology [13,297]. These data point out that alterations in synaptic properties might be good indicators of NMJ denervation and axonal degeneration. Furthermore, previous studies show that, following partial denervation by nerve incision, nerve terminal sprouting is impaired in *SOD1^G93A^* mice prior to disease onset, mainly in axons innervating type IIB muscle fibers [298,299]. However, the resulting denervation is not only due to the impossibility of successful MN sprouting but also impaired TSC process extension that leads to insufficient re-occupation of denervated synaptic sites [99,296,300].

Increasing evidence supports that TSCs are incompatible with synaptic repair in several ALS animal models and patients [301]. TSCs at the NMJ show abnormal morphology with disorganized processes, which are insufficient for sprouting [285]. Indeed, TSCs contribute to terminal degeneration and denervation in ALS, in the *SOD1^G37R^* mouse model, due to improper reinnervation guidance and improper removal of endplate debris [285]. This has also been observed in human TSCs, where extensive processes and intrusion in the synaptic cleft attenuated synaptic transmission in early-stage ALS patients [302]. After experimental partial denervation, TSCs were unable to reoccupy the NMJ and to induce reinnervation in *SOD1^G93A^* mouse model [299]. This process was triggered by the increased macrophage infiltration at the NMJ that attenuated nerve sprouting and synaptic repair [298]. Thereby, despite the presence of positive NMJ repair signs, it does not seem to be efficient enough during disease onset.

Furthermore, TSC synaptic decoding capacity seems to be modified in presymptomatic mice, while NMJ organization appeared normal. Increased activation of TSCs in S and FR NMJs and decreased activation in FF NMJs were observed in presymptomatic mice, where muscarinic AchR dependent activity was higher in both cases [285,296]. These alterations in TSC decoding and excitability lead to an inadequate switch to the phagocytic phenotype. Thus, decreased expression of phagocytic marker *Gal-3* was observed in presymptomatic mouse models [285].

Consequently, the previously described events happen before disease onset and aggravation, reinforcing the “dying back” theory in ALS, with a distal initiator factor at the endplate. Certainly, the lack of a suitable synaptic repair leads to an inadequate environment for nerve terminal sprouting that finally provokes NMJ degeneration and disease progression.

### 5.2. Post-Synaptic Affectation

Both secreted factors in the synaptic cleft and proteins participating in the signaling pathways that regulate nAchR cluster stability are essential for post-synaptic endplate maintenance and NMJ integrity [302]. Alterations in the aforementioned proteins will trigger cluster instability and specialized endplate degradation. Denervation of the NMJ is frequently accompanied by bungarotoxin diffuse labeling, smaller and more fragmented endplates in both ALS patients and animal models [18,87,295,302]. nAchR expression in muscle fibers is restricted to subsynaptic nuclei of innervated muscles, and it is finely regulated by key molecular mechanisms. Amongst others, the proteoglycan Agrin, which is secreted by nerve terminals and TSCs, is known to regulate AchR stability through the LRP4-MuSK receptor complexes [286,303]. Lately, it has been demonstrated that FUS participates in the nAchR expression through the interaction with transcription factors in this pathway, and suggesting that *FUS* mutations-carrying ALS patients and animal models would suffer endplate instability [230]. Thus, myotube and MN co-cultures derived from *FUS*-ALS patient iPSCs showed impaired endplate maturation, and *FUS* mutant mice had decreased endplate surface area [230]. Besides, the neurotrophic factor neuroregulin 1 (*NRG-1*) participates in NMJ maintenance and AchR stability, and Nrg-1-ErbB4 receptor signaling alterations may be involved in the pathogenesis of ALS [304]. In mutant *Sod1* mice, adenoviral overexpression of *Nrg-1* by intramuscular adenoviral injection prevented denervation, activated collateral reinnervation, and stabilized AChR clusters through ErbB receptor activation [305].

As previously indicated, the NMJ allows controlled signaling between muscle and nerve necessary for skeletal muscle function. Indeed, the pivotal role of satellite cells (SC) as secretors of specific factors and attractive-repulsive signals is essential for axonal guidance and restoration of functional innervation during regeneration [16,207]. For instance, the axis HGF/Sema3A and, recently Neuropilin1/Sema3 have been proposed for regenerative motoneuritogenesis, being the skeletal muscle the main source of SEMA3A [16,306]. Strikingly, in a model of peripheral nerve injury that enables NMJ regeneration, increased SC fusion to myofibers was demonstrated in the vicinity of regenerating NMJs [283]. Liu and collaborators have recently shown that NMJ regeneration is impaired in the absence of SCs, and even more, they demonstrate that this SC absence drives age-related NMJ degeneration [307,308]. More specifically, depletion of SCs led to deficits in NMJ reinnervation, reductions in post-synaptic morphology, and loss of post-synaptic myonuclei. Moreover, SC depletion was also associated with reduced myofiber size and further declines in muscle force-generation capacity, indicating the necessity for SC-mediated NMJ regeneration in the regulation of skeletal muscle integrity upon neuromuscular disruption [283].

Remarkably, alterations in these secreted signals, including the aforementioned Sema3A and its receptor Neuropilin 1 affect NMJ stability in *SOD1^G93A^* mice [306]. SEMA3A is a neuro-repellent protein that has the potential to regulate neurite sprouting and seems to be implicated in AChR stability through myogenin [207]. Besides, presymptomatic mutant mice showed alterations in *Sema3a* expression and downregulation of *Sema3a* regulating miR126-5p microRNA expression, which contributed to NMJ dismantling [309]. Meanwhile, miR126-5p overexpression both in vivo and in vitro was sufficient to restore NMJ innervation and functionality. All these mentioned molecules were secreted by other components of the NMJ or the muscle, which at the same time were altered in pre-onset or onset of different ALS patients and animal models symptoms. Therefore, these data remark the importance of correct functioning and communication of NMJ components, and strategies targeting NMJ function and structure preservation should be considered for treatment strategies in ALS.

**Figure 1:**
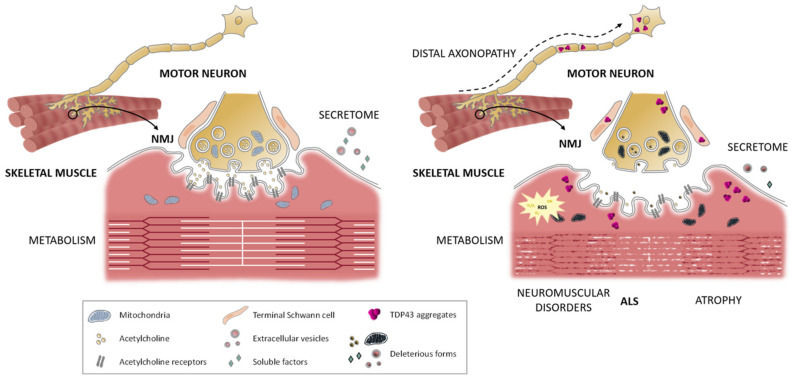
Proposed model for the skeletal muscle as a key contributor on ALS peripheral etiopathogenesis through a “dying back” mechanism. The main components of the neuromuscular junction (NMJ) are depicted for healthy (left) and ALS (right) states, respectively: the pre-synaptic part (nerve terminal), post-synaptic part (motor endplate), and the synaptic cleft in between the two. As illustrated, NMJs in ALS are destabilized, and different components are altered. Indeed, early metabolic and myogenic defects in skeletal muscle trigger mitochondrial dysfunction, oxidative stress, and increased production of reactive oxygen species (ROS). Besides, dysregulated protein homeostasis enhances the presence of cytoplasmic and nuclear aggregates, including TDP-43 inclusions in muscle, that are also present in other cell types, including terminal Schwann cells and motor neurons (MN). Moreover, the skeletal muscle increases extracellular vesicles production (secretome), which may content toxicity and release it to other NMJ cell components. All these muscle alterations result in muscle atrophy and NMJ dismantlement, which are classical hallmarks of ALS. Thereby, altered intercellular communication exacerbates nerve retraction and degeneration of the distal MN ending, which is spread in a retrograde manner (dashed black arrow) into the soma, resulting in progressive MN degeneration.

In summary, a “dying back” phenotype has been described in the peripheral origin of ALS, starting far in the axon with negative retrograde signaling for MN death (Figure 1). While ALS induction in neurons does not recreate ALS, the same implication specifically in muscle recapitulates the disease, including MN degeneration, acknowledging the relevance of the muscle tissue [17,18]. This MN defaults would start at the NMJ in the muscle by perturbation in redox signaling cascades induced by muscle-specific accumulation of mutant SOD1-G93A [92].

## 6. Final Remarks

The attention that muscle has received during many years of ALS research has been limited to the description of muscle as a tissue affected just by the deprivation of neuronal innervation. To date, there is clear evidence that MN degeneration is the result of complex mechanisms and *non-cell autonomous* processes, in which other nerve cell types take chief roles. In this context and taking into account that muscle cells and MNs form one of the most complex communicative assemblies in the body (through the NMJ), it is pertinent to deduce that MN-muscle communication breakdown in ALS does not have to occur solely in response to unilateral transmission of disease, from neurons to muscle, but rather bilateral, which also includes pathological spreading from muscle to neurons. The latter mechanism has given rise to the “dying back” theory of ALS, where the muscle is described as a tissue that can initiate—or at least contribute to—a cascade of pathological events to precipitate a distal onset of MN degeneration (Figure 1). To date, the bulk of studies have provided strong clinical and empirical evidence to support that the skeletal muscle undergoes early changes before MN denervation and onset of ALS symptoms, with the identification of abnormal trophic and metabolic support as key underlying mechanisms. Although the concrete ways through which muscle pathology can affect MNs post-synaptically (i.e., through loss of neuron-specific trophic factors, the release of metabotoxic species, or any other pathological macromolecules) remain unanswered in the context of ALS, the muscle should be at the center of ALS research, as a target tissue to address novel therapies in combination with those oriented to the CNS.

## Figures and Tables

**Table 1:** Common pathological features in motor neurons and the skeletal muscle in ALS.

Common Pathological Features		
	ALS	
*Structural*	Motor neurons	Skeletal muscle
Decreased innervation	+ (axonopathy)	+ + +
NMJ dismantlement (fragmentation & loss)	+ + + (pre-synaptic)	+ + + (post-synaptic)
Reduced mass and strength / atrophy	+ + + (spinal cord)	+ + +
Cytoplasmic & nuclear aggregates	+ + +	+ +
	ALS	
*Functional*	Motor neurons	Skeletal muscle
Metabolic changes	+	+ + +
Oxidative stress	+ + +	+ +
Deregulated nutrient sensing	+	+ +
Loss of proteostasis	+ + +	+ +
Mitochondriopathy	+ +	+
DNA damage	+ +	+
Inflammation	Neuroinflammation	+
Altered intercellular communication	+ +	+
Cellular senescence	+ +	+ (in vitro)
Cell death	+ + +	+ +

Abbreviations: +, low grade; + +, medium grade; + + +, high grade; NA, not applicable.

**Table 2:** Mouse models carrying ALS mutations specifically in the skeletal muscle.

Muscle-Specific ALS Mouse Models						
Mouse Lines	Tissue Specificity	ALS–Gene Regulation	Phenotypic/ Disease Onset	Muscle Phenotype	Neurological Phenotype	References
*Acta1^CRE^;Sod1^flox/flox^ (mSod1KO)*	skeletal muscle (actin -1)	mouse *Sod1* deletion	- Onset: 6–8 months - Survival: >16–17 months	No muscle atrophy Increase weakness Increased regenerative muscle fibers	No NMJ degeneration Not increased ROS production Not decreased mitochondrial ATP production	[88]
*SOD1^G93A^/mIgf*-*1* (muscle rescue)	*SOD1^G93A^* ubiquitous *Igf*-1 in muscle	human SOD1-G93A overexpression and mouse *Igf-1* overexpression	- *G93A*:onset: 90 days survival: <145 days - *G93A/mIgf-1*: onset: 110 days survival: <175 days	Maintenance of muscle integrity	NMJs stabilization Reduced inflammation in the spinal cord Enhanced MN survival	[89]
*MLC/SOD1^G93A^*	skeletal muscle (specific regulatory elements from MLC)	human SOD1-G93A overexpression	- Muscle atrophy: 4 weeks - Functional Performance: 16 weeks - Survival: not indicated	Muscle atrophy Significant reduction in muscle strength Alterations in the contractile apparatus Mitochondrial dysfunction Alteration in fiber type composition and metabolism (fast-to-slow shift)	NMJ dismantlement Microgliosis Hypomyelination in the sciatic nerve No MN loss in ventral spinal cord	[17,90,91,92]
*WT-hSOD1^mus^* *G93A-hSOD1^mus^* *G37R-hSOD1^mus^*	skeletal muscle (chicken -actin)	human *SOD1* overexpression	*Similar phenotype in all cases* - Onset: 8–10 months - Survival: shortened 10–16% (slow disease progression)	Muscle atrophy Limb weakness and paresis Motor deficits Lifespan shortening Adipose tissue waste	*Causes fatal ALS-like* *syndrome:* Severe loss of NMJ Decreased innervation Axonopathy Spinal cord atrophy Loss of MN	[18,87]
*TDP-43 TG*	skeletal muscle (creatine kinase 8)	human *TDP-43* overexpression	- Onset: 36 weeks - Survival: >18 months	Increased serum levels of myogenic enzymes Degenerative myofibers via ER stress Myotoxicity featuring tubular aggregates and TDP-43-positive inclusions	Not described	[93]
*Pax7^IREScre^Tardbp^flox/WT^*	*Pax7* lineage	1 copy of mouse *Tdp-43* deletion in MPCs and progeny	No phenotype in the adult but smaller myofibers after muscle damage-induced regeneration	TDP-43 is essential for skeletal-muscle-cell differentiation in culture and required for skeletal-muscle regeneration	Not described	[94]
*MRL/MpJ*	“super-healing” mouse model with siRNA	mouse *Sod1* or *Cat* depletion (in vitro silencing of MPCs)	Great regenerative capacity for repair of many tissues	Impaired myogenic potential of MPCs A role for antioxidants in muscle repair	Not described	[80]

Abbreviations: SOD1: superoxide dismutase 1; G93A: SOD1 Gly^93^ → Ala described mutation; G37R: SOD1 Gly^37^ → Arg described mutation; *Acta1*: actin α 1 gene; *Igf-1*: insulin-like growth factor isoform 1 gene; MLC: skeletal muscle-specific regulatory elements from rat myosin light chain (MLC)-1/3 locus; *Tardbp*: TAR DNA binding protein gene (TDP-43); *Cat*: catalase gene; *MRL/MpJ*: Murphy Roths Large mouse line; NMJ: neuromuscular junction; MN: motor neuron; MPC: muscle progenitor cells; WT: wild-type; KO: knock-out; h: human; mus: mouse; m: muscle.
